# Variational Autoencoder for Image-Based Augmentation of Eye-Tracking Data

**DOI:** 10.3390/jimaging7050083

**Published:** 2021-05-03

**Authors:** Mahmoud Elbattah, Colm Loughnane, Jean-Luc Guérin, Romuald Carette, Federica Cilia, Gilles Dequen

**Affiliations:** 1 Laboratoire Modélisation, Information, Systèmes (MIS), Université de Picardie Jules Verne, 80080 Amiens, France; jean-luc.guerin@u-picardie.fr (J.-L.G.); r.carette@evolucare.com (R.C.); gilles.dequen@u-picardie.fr (G.D.); 2 Faculty of Science and Engineering, University of Limerick, V94 T9PX Limerick, Ireland; 9926186@studentmail.ul.ie; 3 Evolucare Technologies, 80800 Villers-Bretonneux, France; 4 Laboratoire CRP-CPO, Université de Picardie Jules Verne, 80000 Amiens, France; federica.cilia@u-picardie.fr

**Keywords:** deep learning, variational autoencoder, data augmentation, eye-tracking

## Abstract

Over the past decade, deep learning has achieved unprecedented successes in a diversity of application domains, given large-scale datasets. However, particular domains, such as healthcare, inherently suffer from data paucity and imbalance. Moreover, datasets could be largely inaccessible due to privacy concerns, or lack of data-sharing incentives. Such challenges have attached significance to the application of generative modeling and data augmentation in that domain. In this context, this study explores a machine learning-based approach for generating synthetic eye-tracking data. We explore a novel application of variational autoencoders (VAEs) in this regard. More specifically, a VAE model is trained to generate an image-based representation of the eye-tracking output, so-called scanpaths. Overall, our results validate that the VAE model could generate a plausible output from a limited dataset. Finally, it is empirically demonstrated that such approach could be employed as a mechanism for data augmentation to improve the performance in classification tasks.

## 1. Introduction

Human eyes represent a rich source of information, for communicating emotional and mental conditions, as well as for understanding the functioning of our cognitive system. An eye gaze can serve as an appropriate proxy for learning a user’s attention or focus on context [1]. Therefore, eye-tracking technology has been intensively utilized for studying and analyzing many aspects of gaze behavior.

Eye-tracking refers to the process of capturing, tracking, and measuring the absolute point of gaze (POG) and eye movement [2]. Interestingly, the field of eye-tracking has quite a long history, dating back to the 19th century. The French ophthalmologist Louis Javal, from Sorbonne University, started the initial analysis of gaze behavior in 1878. It is largely acknowledged that Javals’ studies [3,4] laid out the foundations that initially explored the behavior of human gaze in terms of fixations and saccades. Subsequently, Edmund Huey built a primitive eye-tracking tool for analyzing eye movements [5]. More advanced implementations of eye-tracking were developed by [6,7]. Photographic films were utilized to record eye movements while looking at a variety of paintings. The eye-tracking records included both direction and duration of movements.

With technological advances, the field of eye-tracking has evolved towards the nearly universal adoption of video-based methods. Video-based eye-trackers can be classified into the following: (1) video-based tracking using remote or head-mounted cameras and (2) video-based tracking using infrared pupil-corneal reflection (P-CR) [2]. Furthermore, recent developments have discussed the use of virtual reality-based methods for eye-tracking [8]. Eye-tracking has been widely utilized in a multitude of applications for commercial and research purposes. Examples include marketing [9], psychology studies [10], product design [11], and many other applications. 

However, the scarce availability or difficulty of acquiring eye-tracking datasets represents a key challenge, while access to image or time series data, for example, has been largely facilitated thanks to large-scale repositories such as ImageNet [12] or UCR [13]. The eye-tracking literature still lacks such data repositories. In this respect, we explore the use of machine learning (ML) for generating synthetic eye-tracking data in this study. An image-based approach is adopted based on transforming the eye-tracking scanpaths into a visual representation. Using unsupervised learning, a variational autoencoder (VAE) is employed for the generative modeling task. Subsequently, empirical experiments robustly demonstrated that the inclusion of VAE-generated images could improve the performance of models in classification tasks. The primary contribution of this study is claimed to be as exploring a novel application of VAEs in this context. To the best of our knowledge, the proposed approach has not been discussed yet in the literature.

## 2. Background

In this section, we provide a preliminary background on autoencoders and their applications in general. Initially, in the first section, we review the classical autoencoders, mostly used for tasks related to data compression, feature extraction, or denoising. Subsequently, we discuss the VAE approach and its suitability for generative modeling, which is the focus of the present study.

### 2.1. Autoencoders

**Figure 1:**
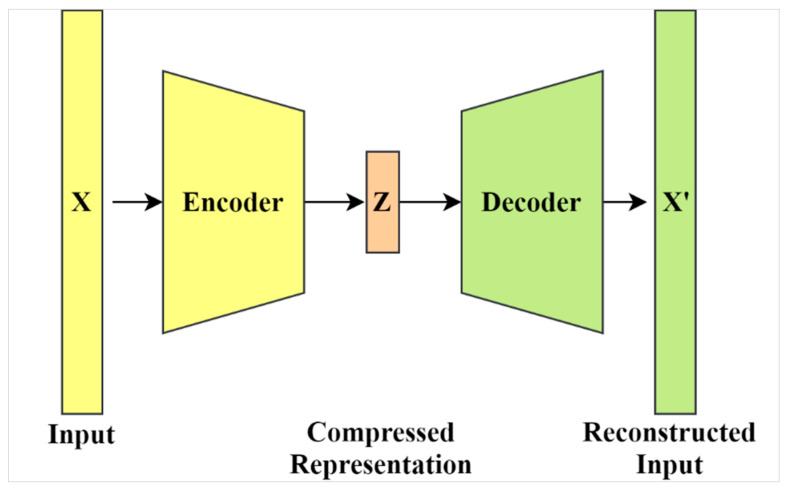
The general architecture of autoencoders.

Generally, autoencoders are considered to be a special implementation of artificial neural networks (ANNs). In contrast to typical ANN applications (e.g., regression and classification), autoencoders are fully developed in an unsupervised manner. Using unsupervised learning, autoencoders learn compressed representations of data, the so-called “codings”. As such, training an autoencoder does not require any label information. The compression and decompression are automatically inferred from data in contrast to being formulated using mathematical equations or hand-crafted features. Figure 1 illustrates the basic architecture of autoencoders including encoding and decoding.

The idea of autoencoders was originally introduced in the 1980s by the parallel distributed processing (PDP) group including Geoffrey Hinton, at the University of California, San Diego. They were generally motivated by the challenge of training a multi-layered ANN, which could allow for learning any arbitrary mapping of input to output [14]. Their work eventually led to the development of the backpropagation algorithm, which has become the standard approach for training ANNs.

There is a variety of valid applications that could be realized by autoencoders. Fundamentally, autoencoders can be used as an effective means to reduce data dimensionality [15,16], whereas codings represent a latent space of significantly lower dimensionality as compared with the original input. Furthermore, autoencoders provide a potent mechanism for feature extraction. More interestingly, they can perform the functionality of generative modeling. The codings learned can be utilized to randomly generate synthetic samples, similar to the original data.

Data denoising is another well-explored application of autoencoders. Denoising autoencoders were first developed by Vincent et al. [17,18]. The basic idea is that the encoder can consider its input as corrupted data, while the decoder attempts to reconstruct the clean uncorrupted version. Therefore, denoising autoencoders can learn the data distribution without constraints on the dimensions or sparsity of the encoded representation. Several studies have experimentally implemented denoising autoencoders in a variety of important applications. For example, denoising autoencoders were successfully applied for speech enhancement and restoration [19,20]. By the same token, a convolutional denoising autoencoder was utilized for reducing the noise in medical images [21].

### 2.2. Variational Autoencoders

Kingma and Welling [22] originally introduced the VAE framework in 2014, which has been considered as one of the paramount contributions for generative modeling or representation learning in general. The VAE approach provided a novel method that jointly coupled probabilistic models with deep learning. In contrast to traditional autoencoders, the fundamental distinction of VAEs is that they learn latent variables with continuous distributions, which has proven to be a particularly useful property while approaching tasks of generative modeling.

**Figure 2:**
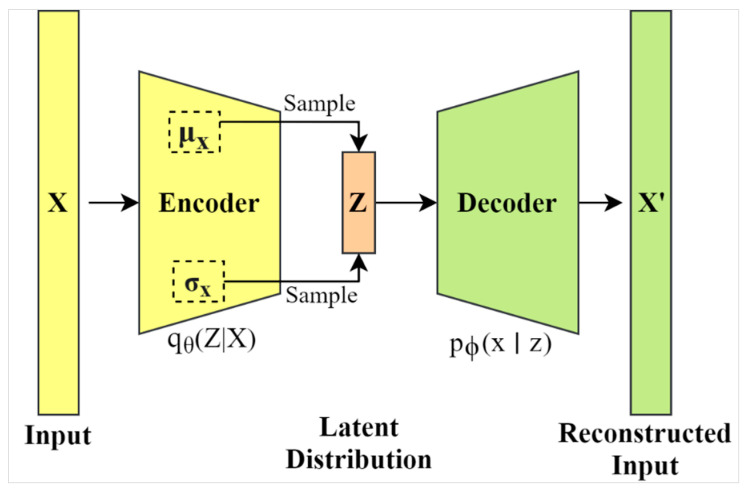
The variational autoencoder (VAE) architecture. X represents the input to the encoder model and Z is the latent representation along with weights and biases (θ).

VAE encoding has been cleverly designed to return a distribution over the latent space rather than discrete values. More specifically, the encoder produces a set of two vectors including a vector of means (μ), and another vector of standard deviations (σ). As such, the VAE attempts to learn the distributions of latent variables based on the mean values and their variances, instead of learning a deterministic mapping, as in traditional autoencoders. Figure 2 shows a sketch of the VAE architecture and it can be observed that the latent dimensional space is stochastic based on the samples of μ and σ values. A comprehensive presentation of the VAE approach goes beyond the scope of this study, however, we recommend the tutorial by Kingma and Welling [23] in this regard.

Since its inception, the VAE approach has been increasingly adopted in a diversity of generative modeling tasks. For example, an RNN-based VAE architecture was implemented for text generation [24]. Likewise, a study [25] developed a hybrid architecture of convolutional neural networks (CNN) and recurrent neural networks (RNN) for text generation as well, while other studies explored the VAE potentials for generating natural images [26,27]. It is also worth mentioning that the generative adversarial network (GAN) by Goodfellow et al. [28] is another popular approach for generative modeling, however, it is not the focus of the present study.

## 3. Related Work

The literature review is divided into two sections as follows: Initially, the first section includes representative studies that implemented VAE-based applications for the purpose of data augmentation or generative modeling in general. The second section reviews contributions that attempted to synthetically generate or simulate eye-tracking output. In this respect, we aim to review approaches that have developed algorithmic models, as well as ML-based methods. The review is selective rather than exhaustive, therefore, it basically aims to highlight representative approaches in this context.

### 3.1. Variational Autoencoder (VAE)-Based Methods for Data Augmentation

The VAE approach has been intensively applied for synthetic data generation, or representation learning in a broader sense. The literature already includes a diversity of studies that made use of VAE-based implementations as a mechanism for data augmentation. 

For instance, a study by [29] explored the beneficial use of VAEs in the case of imbalanced datasets. To this end, they extracted an imbalanced subset of the popular MNIST dataset. The dataset was augmented with synthetic samples generated by a VAE model. Their empirical results demonstrated that the inclusion of VAE-generated samples had a positive impact on the classification accuracy in general. Similarly, a more recent study analyzed the impact of using different augmentation techniques on the model accuracy in supervised ML problems [30]. Their experiments focused on smaller datasets, where the number of samples per class were lower than 1000. The experiments were based on a set of 19 benchmark datasets selected from the University of California Irvine (UCI) data repository [31]. Using VAE and GAN models, their results demonstrated that data augmentation could boost the prediction accuracy by approximately 3%.

From a practical standpoint, the literature includes a broad variety of applications using the VAE approach for augmentation. One recent study used a VAE model to generate traffic data pertaining to crash events [32]. Their work demonstrated how the VAE latent space could be used to generate millions of synthetic crash samples. The use of data augmentation had a significant effect on the model performance since the original dataset was extremely imbalanced. In another application related to acoustic modeling, a VAE-based framework was developed to perform data augmentation and feature extraction [33]. The dataset size of speech corpus could be doubled using the latent variables extracted by the VAE model. Similarly, their results demonstrated that augmentation could improve the performance of speech recognition.

In the context of electroencephalography (EEG), a study used augmentation techniques including VAE [34]. They applied a VAE model to generate realistic features of EEG records, which were used to augment the training data. The empirical results reported a significant improvement in the accuracy of the emotion recognition models. More specifically, the models could achieve up to 10% improvement. Similarly, recent efforts [35] have explored VAE-based methods to augment EEG datasets.

Furthermore, numerous applications have been experimentally studied in the field of medical imaging. For instance, a convolutional VAE model was developed to generate realistic samples of left ventricular segmentations for data augmentation [36]. Another study demonstrated the effectiveness of VAEs for generating synthetic images of clinical datasets including ultrasound spine images and Magnetic Resonance Imaging (MRI) brain images [37]. More complex tasks were approached using VAE-based architectures as well. For example, a VAE-based approach was adopted for the three-dimensional (3D) reconstruction of the fetal skull from two-dimensional (2D) ultrasound planes acquired during the screening process [38]. They developed a VAE model that could integrate ultrasound planes into conditional variables to generate a consolidated latent space. Likewise, a VAE architecture was implemented for the reconstruction of 3D high-resolution cardiac segmentation [39].

### 3.2. Generative Modeling of Eye-Tracking Data

The literature is rife with methods applied for synthesizing or simulating human eye movements, typically captured by eye trackers. The methods can be broadly classified into two schools of thoughts. On the one hand, the early efforts aimed to craft algorithmic models based on characteristics driven from the eye-tracking research. On the other hand, recent studies have been more inclined towards ML-based approaches.

For instance, a study proposed to synthesize the eye gaze behavior from an input of head-motion sequences [40]. Their method was mainly based on the statistical modeling of the natural conjugation of head and gaze movements. Similarly, another study developed a stochastic model of gaze behavior [41]. The synthetic output could be parameterized based on a set of variables such as sampling rate, micro-saccadic jitter, and simulated measurement error.

In a similar vein, there have been plentiful contributions for developing gaze models that can generate realistic eye movements in animations or virtual environments. To name a few, one study implemented statistical models of eye-tracking output based on the analysis of eye-tracking videos [42]. The models were aimed at reflecting the dynamic characteristics of natural eye movements (e.g., saccade amplitude and velocity). Another framework was proposed to automate the generation of realistic eye and head movements [43]. It was basically aimed at separately learning inter-related statistical models for each component of movement based on pre-recorded facial motion data. The framework also considered the subtle eyelid movement and blinks.

Recent experimental studies have been purely ML-based approaches for generating synthetic eye-tracking data. Eye-tracking instruments produce an abundant amount of data including a variety of eye-gaze information. A few minutes of operating time can typically output thousands of records describing gaze positions and eye movements. Hence, ML could be viewed as an ideal path to also develop predictive and generative models. In addition, the emergence of deep learning has played a key role in this regard. Deep learning provides a potent mechanism for learning complex mappings from raw data automatically, avoiding the need for developing hand-crafted features. Implementations of CNNs [44,45] and RNNs [46] have been successfully applied to tackle complex tasks such as computer vision and machine translation.

In this respect, a CNN-based architecture was developed for the semantic segmentation of eye-tracking data [47]. A CNN-based architecture was utilized for the reconstruction and generation of eye movement data. Another study proposed a convolutional-recurrent architecture, named “PathGAN” [48]. On the basis of adversarial learning, the PathGAN framework presented an end-to-end model for predicting the visual scanpath. In another application, a real-time system for gaze animation was developed using RNNs [49]. Motion and video data were both used to train the RNN model, which could predict the motion of body and eyes. The data were captured by a head-mounted camera.

Moreover, long short-term memory (LSTM) architectures have been developed to generate synthetic eye-tracking data, for instance, a sequence-to-sequence LSTM-based architecture was developed to this end [50]. More recently, another recent study proposed a text-based approach using an LSTM implementation [51]. The key idea was to represent eye-tracking records as textual strings, which described the sequences of fixations and saccades. As such, they could apply methods from the natural language processing (NLP) domain to transform and model eye-tracking sequences, while an LSTM model was employed for the generative modeling task.

## 4. Data Description

The dataset under consideration was collected as part of our earlier work related to the detection of autism using eye-tracking [52]. Abnormalities of eye gaze have been largely identified as the hallmark of autism spectrum disorder (ASD) [53]. As such, eye-tracking methods are widely utilized in this context.

The dataset was originally constructed as follows: A group of 59 children participated in a set of eye-tracking experiments. The age of participants ranged from 3 to 12 years old. The participants were grouped into two cohorts as follows: (i) typically developing (TD) and (ii) ASD. The participants engaged in watching a set of photographs and videos, which included social cognition scenarios according to their age, to stimulate the viewer’s gaze. The average period of time of each eye-tracking experiment was about 5 min.

The experiments were conducted using an eye-tracker by SensoMotoric Instruments (SMI) (Teltow, Germany) with 60 Hz sampling rate. The eye-tracking device captured three categories of eye movements including fixations, saccades, and blinks. A fixation describes a brief period of gaze focus on an object, which allows the brain to perform the process of perception. The average timespan of fixations is estimated to be around 330 ms [54]. Saccades include rapid and short eye movements that perform constant scanning and consist of quick ballistic jumps of 2° or longer, with an average duration of about 30–120 ms each [55]. The output of a sequence of fixations and saccades is defined as a scanpath.

A set of 25 eye-tracking experiments was conducted to produce the output dataset. The dataset was stored in multiple CSV files, which collectively included more than 2M records. For the purpose of demonstration, Table 1 provides a few eye-tracking records as captured by the eye-tracking device which describe the category of movements and the POG coordinates over the experiment runtime. Specifically, each row represents a point in the experiment timeline, where the eye-tracking timing was approximately 20 ms. Due to limited space, many other variables had to be excluded from the table (e.g., pupil position and pupil size).

## 5. Data Transformation

Data transformation was of paramount importance since the eye-tracking output was obviously high-dimensional. Therefore, the aim was to transform the eye-tracking data into a representation more amenable for ML. The basic idea of our approach was to produce a compact image-based format of eye-tracking scanpaths. This section elaborates on the data transformation procedures.

**Figure 3:**
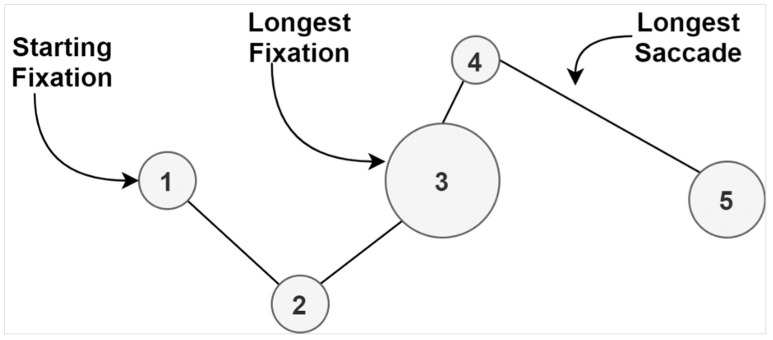
Eye-tracking scanpath [56].

Initially, it is important to clearly define a scanpath, which is the building block of data. A scanpath represents a sequence of consecutive fixations and saccades as a trace through time and space that may overlap itself [56]. The term was first brought into use by Noton and Stark in 1971 [57]. Scanpaths are commonly utilized in eye-tracking applications as a practical means to depict gaze behavior in a visual manner. Figure 3 represents an example of a basic scanpath, which includes a small number of fixations and saccades. The fixations are shown as circles, while the saccades represent the lines connecting those fixations. The diameter of fixations indicates the duration, and the lengths of lines represent the continuation of saccades.

As we previously mentioned, our approach was based on transforming eye-tracking output (i.e., scanpaths) into an image-based format. Our representation of scanpaths follows on the core idea of visualizing fixations and saccades. Moreover, we aimed to visually encode the dynamics of gaze using color gradients. Given the coordinates/time information, we were able to calculate the velocity of gaze movement. Using the grayscale spectrum, the color values were tuned based on the magnitude of velocity with respect to time. The visualizations were produced using Matplotlib library [58]. A comprehensive presentation of that part is elaborated in our earlier work [52].

**Figure 4:**
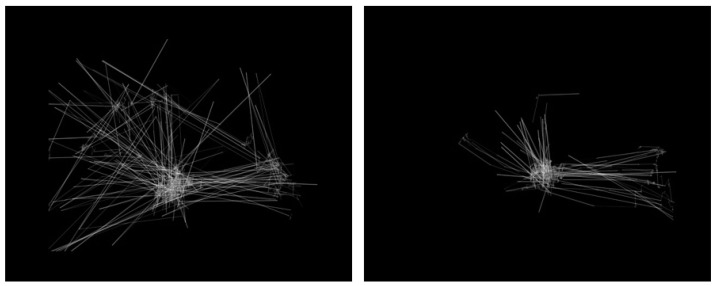
Visualization of eye-tracking scanpaths [52]. The **left**-sided image represents an autism spectrum disorder (ASD) sample, while the **right**-sided image represents the typically developing (TD).

The outcome of the transformation process was an image dataset containing more than 500 images. Specifically, 328 images related to the TD participants, and another 219 images for the ASD-diagnosed. The default image dimensions were set as 640 × 480. The dataset along with its metadata files have been made publicly available on the Figshare repository [59]. Figure 4 presents two examples from the dataset.

## 6. Experiments

The empirical ML experiments consisted of two stages. The initial experiments included the generative modeling of eye-tracking scanpaths. This included the design and implementation of the VAE model. Subsequently, the other stage of our experiments included the development of a classification model to predict ASD based on the scanpath images. The original dataset was augmented using the VAE-generated images produced earlier. The experiments basically aimed to explore the impact of data augmentation on the model performance.

### 6.1. Preprocessing

Initially, a set of preprocessing procedures was applied to simplify the representation of scanpath images. First, the images were cropped in order to remove the blank background. The cropping was based on finding the contour area around the scanpath, which would minimize the background. The cropping was facilitated by using functions from the OpenCV 4.5 library [60].

Second, the images were scaled down to dimensions of 100 × 100. Resizing the images generally heled to reduce the data dimensionality by decreasing the number of features under consideration. Furthermore, it was clear that high-resolution images were not necessary in our case at all, whereas the scanpaths basically represented geometric visualizations rather than natural images.

### 6.2. VAE Experiments

A convolutional VAE was implemented to investigate the latent representation of scanpath images. The VAE model was designed based on a simple symmetric design, where both the encoder and decoder were composed of two convolutional layers, followed by a single fully connected layer. The input images of (100 × 100) dimensions were encoded into a set of (128 × 1) latent variables, which followed a continuous distribution. The mean and variance of distributions were also estimated by the encoder model.

**Figure 5:**
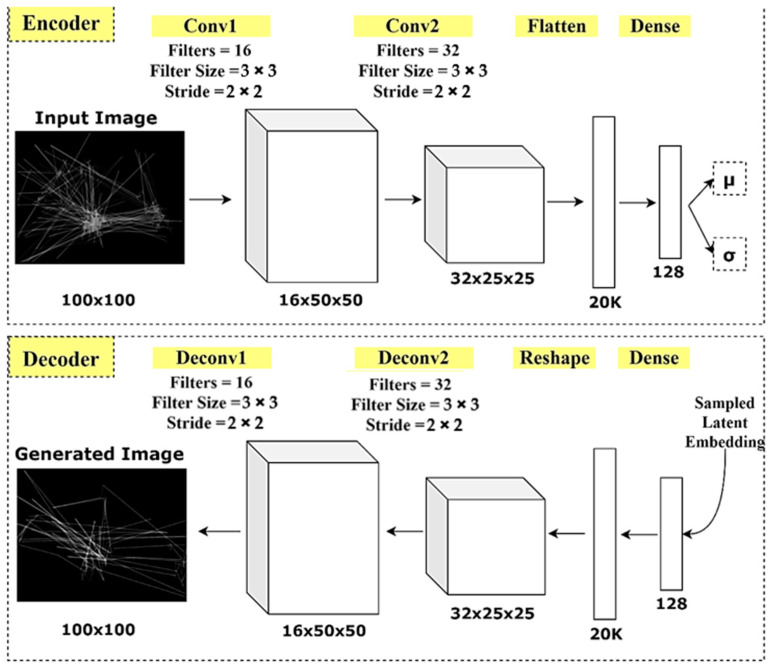
The architecture of the VAE model.

The decoder model was a “flipped” version of the encoder. Inversely, a fully connected layer followed by two deconvolutional layers were stacked in the decoder model. The decoder’s output is a reconstructed scanpath image. Figure 5 shows a sketch of the VAE model architecture.

**Figure 6:**
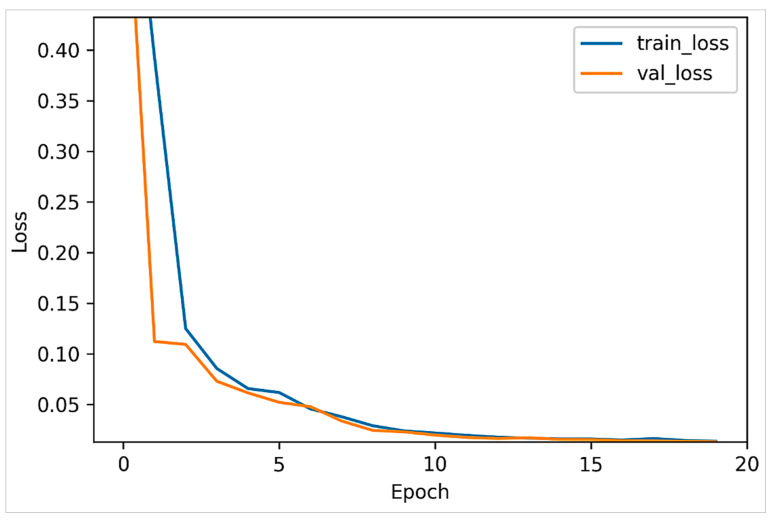
VAE loss in training and validation sets (ASD-diagnosed set).

**Figure 7:**
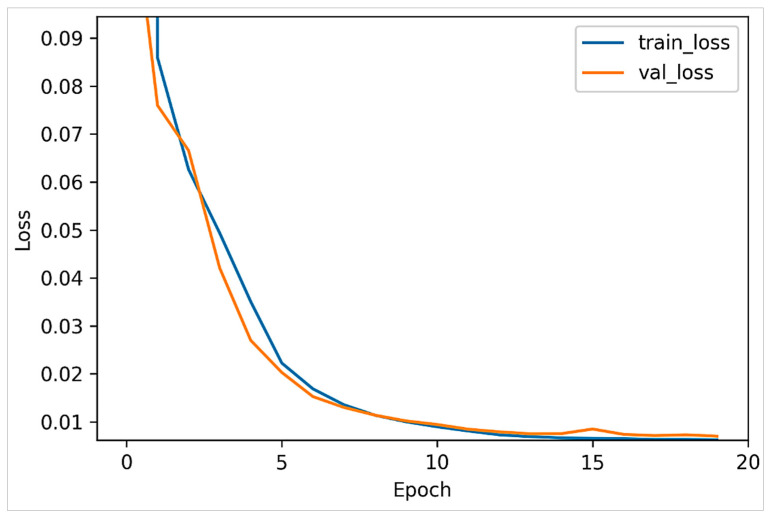
VAE loss in training and validation sets (TD set).

Specifically, two versions of the VAE model were trained using the ASD and TD samples separately. As such, the dataset was initially split into two partitions, where each partition included exclusively a single category of samples. Each VAE model was trained over 20 epochs, and 30% of the dataset was used for validation. Figure 6 and Figure 7 plot the model loss in the training and validation sets for the positive and negative datasets, respectively. It can be observed that the VAE models both largely converged after 10 epochs.

**Figure 8:**
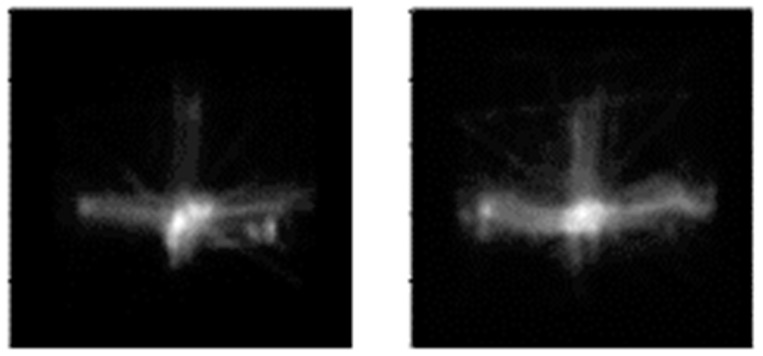
Examples of VAE-generated images.

The model was implemented using Keras [61] with the TensorFlow backend [62]. Eventually, the VAE models were used to generate synthetic scanpath images. Around 300 images were generated for each category. Figure 8 demonstrates two sample images generated by the VAE model.

### 6.3. Classification Experiments

This part aims to investigate the impact of data augmentation on the performance of classification models. Specifically, we compared the model performance before and after the inclusion of the VAE-generated images as part of the training set.

A CNN model was implemented for the classification experiments. The model was composed of four convolutional layers. Each convolutional layer was followed by a max-pooling operation. Eventually, the model included two fully connected layers. A Rectified Linear Unit (ReLU) was used as the activation function in all layers. The dataset was partitioned into training and test sets based on a three-fold cross-validation. The experiments included two scenarios. On the one hand, the model was trained without including the synthetic images. On the other hand, the model was re-trained after the inclusion of the VAE-generated images in the training set. However, the test set always included samples from the original dataset in both scenarios.

**Figure 9:**
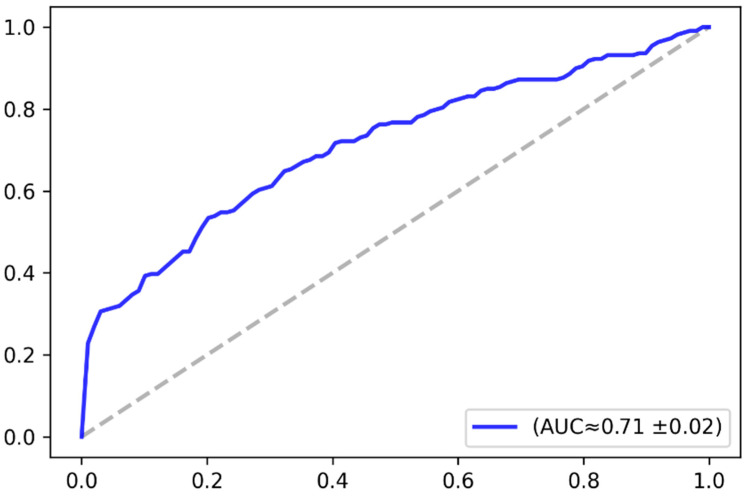
Receiver operating characteristics (ROC) curve-baseline model (no data augmentation).

**Figure 10:**
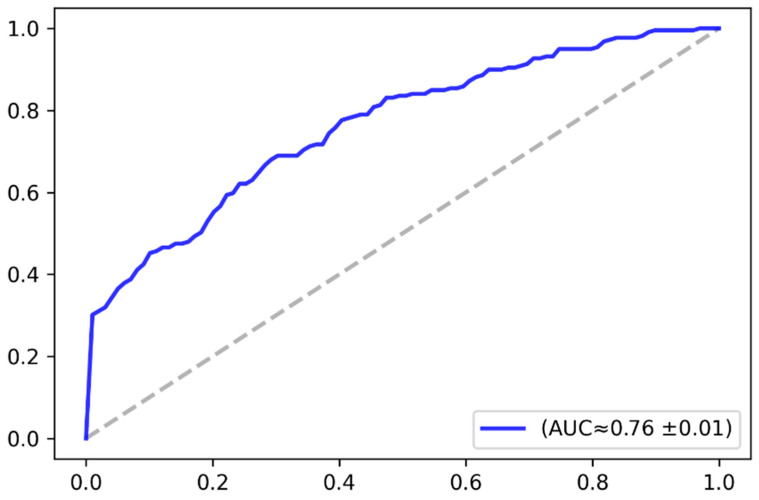
ROC curve after applying VAE-based data augmentation.

The classification accuracy was analyzed based on the receiver operating characteristics (ROC) curve. The ROC curve plots the relationship between the true positive rate and the false positive rate across a full range of possible thresholds. Figure 9 plots the ROC curve in the baseline case (i.e., without augmentation), while Figure 10 plots the ROC curve in case of applying the VAE-based data augmentation, as previously explained. The figures give the approximate value of the area under the curve and its standard deviation over the three-fold cross-validation. The AUC-ROC values demonstrate that the model performance consistently improved after augmenting the dataset with the synthetic images. Table 2 elaborates further on the model performance in terms of accuracy and AUC-RCO as well. The results demonstrated that the overall classification accuracy was improved by approximately 3%.

The training process was completed over 10 epochs using an Adam optimizer [63] with its default parameters. The dropout technique [64] was applied, which helped to minimize the possibility of overfitting. The classification models were implemented using Keras [61] with the TensorFlow backend [62]. Other libraries were certainly useful including Scikit-Learn [65] and NumPy [66]. All experiments were run on the Google Cloud platform using a VM containing a single P-100 Nvidia GPU, and 25 GB RAM.

## 7. Conclusions

The application of data augmentation has been recognized to generally improve the prediction accuracy of image classification tasks [67]. Earlier studies [68,69] sought to generate synthetic images by applying various transformations. Examples included geometric transformations such as random translation, zooming, rotation, flipping, or other manipulations such as noise injection. More recent studies have aimed to utilize the state-of-the-art approaches for generative modeling. In this respect, VAE-based and GAN-based implementations are being increasingly adopted for data augmentation tasks.

In this regard, the results of the present study support the potential of VAE models to perform as an effective mechanism for data augmentation. We demonstrated how a VAE-based approach could be used to generate synthetic eye-tracking data. The mainstay of our approach is the visual representation of eye-tracking data, which allowed for an amenable representation for training the VAE model.

The empirical results clearly confirmed the positive impact of data augmentation on the model’s performance. The classification accuracy could be improved by augmenting the training set with the VAE-generated images. It is proposed that the lack of open access eye-tracking datasets could make our approach attractive for further investigation. For instance, VAE models can serve as an alternative method for data generation in a wide range of eye-tracking applications.

## Figures and Tables

**Table 1:** Samples of the of eye-tracking dataset.

Recording Time [ms]	Category of Movement	Coordinates of POG (X,Y) [px]	Diameter of Pupil (Right, Left) [mm]
8,075,764.426	Fixation	784.4646, 707.7583	4.7591, 4.6711
8,075,808.431	Saccade	784.6325, 707.6475	4.642, 4.6457
8,075,852.429	Fixation	784.4073, 707.5976	4.7215, 4.6723
8,075,896.554	Saccade	784.5244, 708.0931	4.7478, 4.6683
8,075,940.549	Saccade	784.2977, 708.3432	4.6815, 4.6917

**Table 2:** Model performance after data augmentation.

Model	AUC-ROC	Avg. Accuracy
Baseline case (no augmentation)	0.71	67%
With VAE-based augmentation	0.76	70%

## Data Availability

The image dataset is publicly available on the Figshare data repository, https://figshare.com/s/5d4f93395cc49d01e2bd (accessed on 2 May 2021).
